# Climate change jeopardizes the persistence of freshwater zooplankton by reducing both habitat suitability and demographic resilience

**DOI:** 10.1186/s12898-018-0158-z

**Published:** 2018-01-24

**Authors:** Tom Pinceel, Falko Buschke, Margo Weckx, Luc Brendonck, Bram Vanschoenwinkel

**Affiliations:** 1 Animal Ecology, Global Change and Sustainable Development, KU Leuven, Charles Deberiotstraat 32, 3000 Louvain, Belgium; 2 Centre for Environmental Management, University of the Free State, P.O. Box 339, Bloemfontein, 9300 South Africa; 3 Water Research Group, Unit for Environmental Sciences and Management, North-West University, Private Bag X6001, Potchefstroom, 2520 South Africa; 4 Community Ecology Laboratory, Department of Biology, Vrije Universiteit Brussel (VUB), Pleinlaan 2, 1050 Brussels, Belgium

**Keywords:** Bet hedging, Dormancy, Diapause, Environmental change, Life history

## Abstract

**Background:**

Higher temperatures and increased environmental variability under climate change could jeopardize the persistence of species. Organisms that rely on short windows of rainfall to complete their life-cycles, like desert annual plants or temporary pool animals, may be particularly at risk. Although some could tolerate environmental changes by building-up banks of propagules (seeds or eggs) that buffer against catastrophes, climate change will threaten this resilience mechanism if higher temperatures reduce propagule survival. Using a crustacean model species from temporary waters, we quantified experimentally the survival and dormancy of propagules under anticipated climate change and used these demographic parameters to simulate long term population dynamics.

**Results:**

By exposing propagules to present-day and projected daily temperature cycles in an 8 month laboratory experiment, we showed how increased temperatures reduce survival rates in the propagule bank. Integrating these reduced survival rates into population models demonstrated the inability of the bank to maintain populations; thereby exacerbating extinction risk caused by shortened growing seasons.

**Conclusions:**

Overall, our study demonstrates that climate change could threaten the persistence of populations by both reducing habitat suitability and eroding life-history strategies that support demographic resilience.

**Electronic supplementary material:**

The online version of this article (10.1186/s12898-018-0158-z) contains supplementary material, which is available to authorized users.

## Background

Climate scenarios predict increased temperatures and environmental variability for many regions across the globe [1, 2]. These changes are likely to increase species extinction rates [3, 4]. Organisms that depend strongly on short windows of rainfall and suitable temperatures to complete their life cycles may be especially vulnerable to climate change. They will be exposed to shortened growing seasons, which impose more stringent time constraints on maturation and reproduction, reduce population growth rates and increase extinction risks [5–7].

Many organisms from temporary habitats, such as desert annuals or temporary pool zooplankton, have dormant life stages (seeds or eggs), known as propagules, that can withstand harsh conditions including desiccation and survive extended dry periods as part of a propagule bank [8, 9]. The propagule bank spreads recruitment over multiple potential growing seasons through long term dormancy and delayed development, thereby buffering populations against unfavorable growing seasons and demographic catastrophes [10, 11]. Both empirical and modelling studies demonstrate that propagule banks are crucial for the resilience of populations, especially when growing seasons are often unsuitable for successful reproduction [7, 9, 12].

The propagule bank can only promote population resilience when two crucial assumptions are met. First, a given fraction of propagules must survive desiccation until the next suitable growing season [9]. Thus, propagules must be able to remain dormant for long periods without dying and their dormancy should be lifted by cues of favorable conditions [13–15]. Second, all propagules should not hatch or germinate simultaneously during any given reproductive window as this could lead to a demographic catastrophe if the window is too short for successful reproduction. The fractions of propagules that resume development or remain dormant are probably important determinants of long term population fitness [7, 16, 17], but empirical evidence supporting this is sparse [11]. Combined, propagule survival and development rates buffer populations against environmental stochasticity.

Although it has been suggested that propagule banks will be vital for organisms such as freshwater zooplankton to maintain positive long term population growth in the face of climate change [12, 18, 19], little research exists on the possible effects of climate change on propagule banks. For instance, it is mostly unknown whether propagule survival would be affected by realistic changes in current temperatures under climate change. If this is the case, climate change could threaten the very mechanisms needed for demographic resilience. However, thus far, there are no studies that have investigated the long term effects of changes to current temperature cycles on the survival of dormant propagules of plants or animals, let alone the consequences for population growth rates. In this context, demographic models that can incorporate experimentally determined responses of life history traits and link this to fitness, may have an important role to play and can contribute to more realistic estimates of the responses of populations to climate change.

Here, we used an animal with dormant propagules as a model system and performed a long term experiment to measure the impact of increased temperature on the survival and hatching of propagules. We then fed these measured life-history parameters into a recently developed matrix population model [12] and examined how they affect the long term persistence of populations. As model organism, we used fairy shrimps (Crustacea, Branchiopoda, Anostraca), which are common inhabitants of temporary aquatic habitats on all continents. They are dominant filter feeders and strong competitors and their biomass is important as a food source for higher trophic levels [20]. Our model species is *Branchipodopsis wolfi* Daday 1910, a fairy shrimp from small temporary pool habitats in South Africa [21, 22]. These specific habitats already experience extreme climate conditions and propagules in sun-exposed sediment regularly confront temperatures exceeding 45 °C. Climate scenarios project a further increase in mean air temperature of approximately 4 °C by 2070 [1, 28]. Since this species may already be approaching the physiological limits of drought tolerance, it is likely to be more vulnerable to the effects of increased temperatures under climate change than species that experience less extreme conditions.

During an 8-month laboratory experiment, we exposed *B. wolfi* propagules to three different temperature treatments: a present-day temperature cycle, a future temperature cycle as expected under climate change and a constant 18 °C treatment. By performing this trial both with recently produced (2 months old) and older propagules (12 months old), we investigated whether age influences temperature sensitivity. To follow-up on changes in survival rates and hatching fractions of eggs that were exposed to different treatments, we quantified these parameters every 8 weeks in a common garden laboratory hatching experiment.

We hypothesize that anticipated increases in temperature will reduce propagule survival and that older eggs will be more sensitive to increased temperatures than younger eggs due to energy depletion. Furthermore, we propose that hatching fractions will increase with higher temperatures because increased energy consumption and subsequent energy depletion may release propagules from dormancy prematurely. Finally, we expect that combining the measured temperature responses with other life history parameters in a matrix population model will demonstrate that long term population growth rates will decline if temperatures increase, thereby exacerbating the risk of extinction.

## Methods

### Study species

The fairy shrimp *B. wolfi* is one of the dominant crustacean species in temporary aquatic ecosystems in Southern Africa, from large pans to small rock pools. In this study, we used populations from rock pools on the summit of Korannaberg mountain (Free State Province, South Africa), S 28° 51′13″, E 27° 13′51″ (Additional file 1), which have been the subject of several ecological studies (e.g. [12, 21–24]). Adapted to short-lived temporary waters, *B. wolfi* individuals generally hatch within the first days of a new inundation and reach maturity within just 6 days after pool filling. Subsequently, they reproduce sexually to produce between 20 and 30 dormant eggs per day [25, 26]. Like the propagules of many zooplankton species, the eggs are highly resistant to adverse conditions, including extreme temperatures and drought, and can remain viable in a dormant state for many years [25].

### Sediment collection and egg production

Dry sediment with *B. wolfi* propagules was collected following the protocol by Vanschoenwinkel and colleagues [26] from nine rock pools representative of the whole cluster of 44 pools. Care was taken to select different types of pools including small, large, deep and shallow pool basins.

To start-up populations in the Laboratory of Aquatic Ecology, Evolution and Conservation, KU Leuven, 150 g of dry sediment from each population was transferred to an 8 L plastic aquarium and inundated with “Environmental Protection Agency” (EPA) medium with a conductivity of 50 µs cm^−1^ (distilled water with 0.00033 mol L^−1^ NaHCO_3_, 0.000098 mol L^−1^ CaSO_4_·H_2_O, 0.00014 mol L^−1^ MgSO_4_, 0.000015 mol L^−1^ KCL) [27]. Aquaria were aerated and kept under a 12 h light:dark cycle (white light, full spectrum, 4000 lx, lamp type Osram L 8W/640; Osram, Rotterdam, The Netherlands) in a temperature controlled incubator at 18 °C. The medium was replenished daily to maintain constant water levels and hatchlings were fed ad libitum with the unicellular alga *Scenedesmus obliquus* (± 10^6^ cells mL^−1^). Before reaching maturity, all *B. wolfi* individuals were isolated from the rest of the zooplankton community, transferred to clean aquaria of the same volume and kept under identical rearing conditions. Subsequently, the populations were bred for two generations under these common garden conditions to minimize long lasting maternal effects resulting from differences in the environmental conditions in the pools of origin.

Dormant eggs were harvested using a glass pipette and transferred to petri-dishes in temperature-controlled incubators at 18 °C and under a 12 h light:dark regime for dehydration. *B. wolfi* eggs of two age classes were used as starting material for the long term experiment; ‘old eggs’, which had been stored for about 12 months and ‘young eggs’, which were 2 months old. Both batches of old and young eggs were composed of a random mixture selected in equal proportions from all nine laboratory populations.

### Temperature cycles

Hourly temperature data for the Korannaberg region were collected between 01 January 2012 and 31 December 2014 (Centre for Environmental Management, University of the Free State, Bloemfontein, South Africa). Based on these measurements, we calculated the average temperature for each hour of the day, with separate values calculated for each month of the year. We converted these ambient air temperatures to the temperatures actually experienced in the sun-exposed sediment of a rock pool at a depth of 0.5 cm using an hourly conversion factor (Additional file 2). The hourly conversion factors were determined by calculating the ratio between the temperature that was measured at 0.5 cm depth in sun-exposed sediment of a rock pool and the air temperature at that moment.

Climate models predict a temperature increase of approximately 4 °C for the Korannaberg region by 2070 [28]. Based on this prediction and the ambient-sediment conversion factor, we reconstructed daily temperature cycles that are anticipated under climate change in 2070 (Additional file 2). In addition to current and cycles of expected future temperatures, we included a third treatment at a constant 18 °C, which is the temperature that results in optimal egg survival and subsequent hatching under laboratory conditions (unpublished data).

### Incubation experiment, egg survival and hatching trials

A total of 2448 ‘old’ and 2448 ‘young’ viable *B. wolfi* eggs were divided randomly over the three temperature conditions. Since the laboratory experiment was initiated in the month of October, we opted to use the October temperature conditions as a starting-point (Additional file 2). Intact dry eggs (i.e. eggs with no external signs of degradation) were placed individually into the empty wells of a 24-well polystyrene multi-well plate. *B. wolfi* eggs rapidly disintegrate when the embryo is dead, so external features give a reasonable indication of viability. Each egg was assigned to a separate well to avoid any potential interference among eggs and to ensure statistical independence. Still, we chose to include ‘plate identity’ as a random factor in our analyses to correct for any potential plate-effects.

Plates were randomly positioned in temperature controlled incubators, which were programmed to maintain the desired temperature regimes, under a 12 h light:dark cycle and a constant relative humidity of 70%. To minimize any confounding effects of the different incubators, temperature regimes were re-divided across incubators and plates were randomly repositioned within incubators three times during the experiment. Furthermore, plates were randomly repositioned within each incubator on a weekly basis to exclude position effects.

We investigated the effects of the different temperature regimes on survival rates and hatching fractions of both old and young eggs after 8, 16, 24 and 36 weeks. Hatching fractions were established during common garden experiments under optimal hatching conditions (cf. [23]). During each hatching experiment, seven 24-well plates (i.e. 168 eggs) were taken from each of the six conditions and each well was inundated with 2 mL of EPA medium with a conductivity of 50 μS cm^−1^. The plates were randomly positioned within an incubator at 18 °C and under continuous light (white light, full spectrum, 4000 lx). Hatching was evaluated under a light microscope with a 40× magnification at 12 h intervals until no further hatching was observed for 36 h. At the end of each hatching experiment, the viability of every individual egg was checked according to the protocol of Pinceel and colleagues [24] by removing the egg shell with a fine pair of tweezers and evaluating the embryo under a light microscope. Based on this, the number of dead eggs was subtracted from the original number of eggs before calculating hatching fractions.

### Statistical analyses

All analyses were performed in R v. 3.3.1 (R Development Core Team, Vienna, Austria, 2014) and the ‘lme4’ (version 1.1.10) and ‘multcomp’ (version 1.4.6) packages. We tested for effects of temperature regime on egg survival and hatching fraction using generalized linear mixed models (GLMM) with a binomial error distribution and corresponding *logit* link function since both egg survival (dead or alive) and hatching (no hatch or hatch) were measured as the binary response of individual eggs. In a first GLMM, ‘plate identity’ was included as a random factor, ‘temperature regime’ (present-day cycle, future cycle, constant 18 °C), ‘incubation period’ (8, 16, 24 or 36 weeks) and ‘egg age’ (old or new) as fixed categorical predictors and the binary variable ‘egg survival’ (dead or alive) as response variable. To test for effects of the temperature treatments on hatching, we used a second analogous GLMM with ‘egg hatching’ as a binary response variable. The models were built using the glmer function in the lme4 package for linear mixed effects models. We used likelihood ratio tests (LRT) to test the significance of the main effects. We did this by comparing models with one main effect (the one of interest) excluded to a model with all main effects and the random effect via the ‘drop1’ function. In addition, we performed Tukey post hoc tests using the ‘glht’ function in the ‘multcomp’ package to investigate pairwise differences in survival among resting eggs from the three different temperature regime treatments. Since different plates of eggs were removed from the incubators at each time step to test viability and hatching, there was no need for a repeated measures design with incubation period as a random factor.

### Matrix population model

We estimated the impact of measured changes in survival of *B. wolfi* eggs on population demographics using a stochastic matrix population model [12]. This model was developed using realistic life-history parameters to simulate population growth rates and extinction risks across 1000 inundations from eight hydrological regimes (median inundation lengths from 5 to 12 days) that closely match those found in nature [12, 29]. The model comprises two life stages, one corresponding to two age classes to account for age-specific trait values: (1) eggs produced during the previous inundation, N_0_, (2) older eggs in the egg bank, N_1_, and (3) individuals from the active population in the water column, N_2_. Detailed information on the modelling procedure is included in Additional file 3 and in Pinceel et al. [12]. The selection of egg survival parameter values is motivated further in Additional file 4.

## Results

**Fig. 1:**
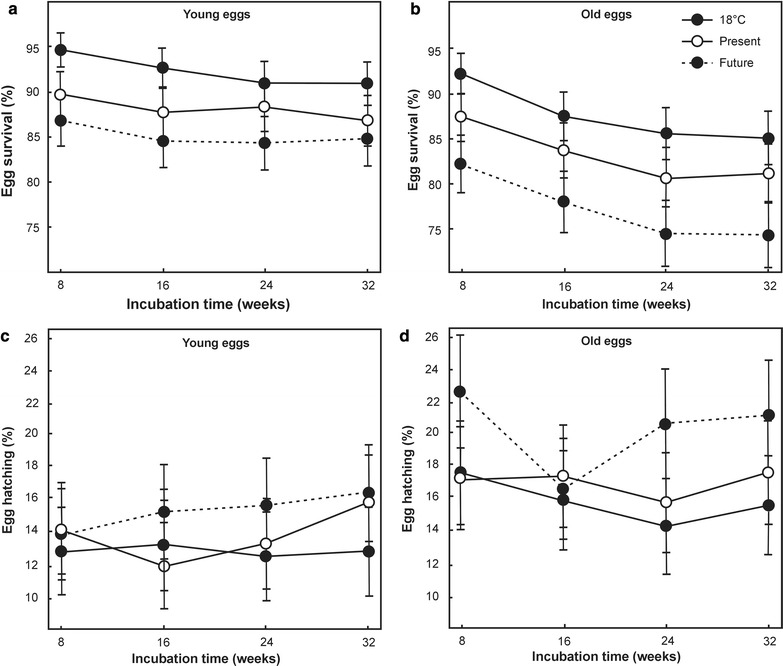
Surviving and hatching fractions of *Branchipodopsis wolfi* eggs were determined every 8 weeks during an 8 month laboratory experiment under three different temperature treatments; a present-day cycle (Present), an expected future cycle (Future) and constant 18 °C (18 °C). Both cycles represent daily temperature fluctuations that were calculated based on the average temperature for each hour of the day, with separate values calculated for each month of the year. The experiment included both young and old eggs that were aged 2 and 12 months at the start of the experiment, respectively. Survival of **a** young and **b** old *B. wolfi* eggs decreased significantly with increased temperatures and incubation time. Hatching fractions of **c** young eggs were not significantly impacted by temperature or incubation time while **d** hatching of old eggs increased significantly in the future climate treatment. Error bars represent standard errors

According to the GLMM, egg survival rates differed between the temperature regime treatments (χ^2^ = 43.186, P < 0.001), survival rates of young eggs were significantly higher than those of old eggs (χ^2^ = 28.570, P < 0.001) and survival decreased significantly with increasing incubation time (χ^2^ = 11.795, P < 0.001) (Fig. 1a, b; Additional file 4). As expected from our first hypothesis, Tukey post hoc tests showed that the future climate treatment reduced egg survival significantly more than the present-day (P = 0.015) and constant 18 °C treatments (P = 0.008). Combined, these findings support the assumption that prolonged exposure to hot conditions will increase egg mortality.

Contrary to our second hypothesis, there was no evidence that higher temperatures lead to increased egg hatching fractions (χ^2^ = 5.531, P = 0.063) and hatching fractions did not increase with increasing incubation time (χ^2^ = 0.027, P = 0.869). However, young eggs were less likely to hatch than old eggs (χ^2^ = 8.687, P = 0.003) (Fig. 1c, d; Additional file 4).

**Fig. 2:**
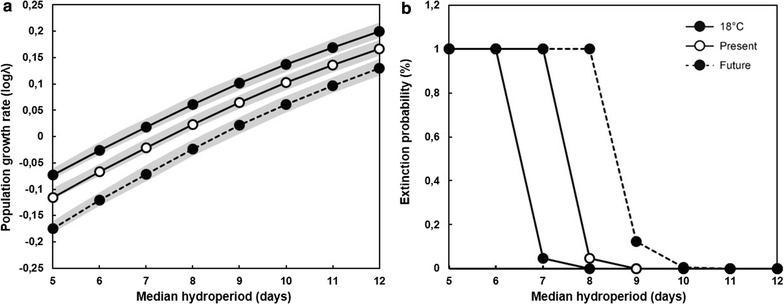
Surviving fractions of *Branchipodopsis wolfi* eggs were determined during an 8 month laboratory experiment under three different temperature treatments; a present-day cycle (Present), a future cycle (Future) and constant 18 °C (18 °C). Both cycles represent daily temperature fluctuations that were calculated based on the average temperature for each hour of the day, with separate values calculated for each month of the year. When survival parameters of old and young eggs are set to the average values measured over the four time points under the expected future temperature treatment, matrix population models indicate that **a** the median hydroperiod required for positive population growth increases and that also **b** the extinction risk of populations in pools with a certain median hydroperiod increases. The grey bands represent standard errors of population growth rate estimates

After incorporating average egg survival, as measured in the current temperature treatment, in the matrix population model, simulations indicated that *B. wolfi* can maintain positive long term population growth rates in pools with a median inundation length of 8 days (Fig. 2a, see Additional file 3 for a worst-case scenario). However, in line with our third hypothesis, when egg survival was set to the values measured in the expected future temperature treatment, the minimum required median inundation length increased to 9 days (Fig. 2a).

Our simulations show that under current temperature conditions extinction risk increases from 0.04 to 4.7% as the median inundation length decreases from 9 to 8 days. In contrast, a similar decline in median inundation length under climate change temperatures will inflate the extinction risk from 12.3 to 100% (Fig. 2b, see Additional file 3 for a worst-case scenario).

## Discussion

Before the end of this century, climate scenarios project that ambient temperatures will have increased up to 4 °C across semi-arid regions; including large parts of South Africa [1, 28]. These changes are expected to alter the hydrological regimes of temporary aquatic ecosystems considerably [29, 30], which compromises their suitability as habitats for aquatic species. In this study, we demonstrated how rising temperatures will not only threaten the persistence of aquatic species by reducing habitat suitability, but by also compromising a common life-history strategy that supports demographic resilience. Once fed into a matrix population model, our findings showed that higher temperatures reduce the survival rates of dormant propagules in a way that jeopardizes the long term persistence of these populations and may lead to the extinction of vulnerable populations.

The susceptibility of zooplankton populations to drying in temporary aquatic ecosystems is well known [5, 6, 12]. Rather than decreasing gradually with decreasing inundation length, our simulations suggest that the probability of *B. wolfi* population persistence depends on a specific threshold, which represents a tipping-point for long term survival. When egg survival rates are based on those under present-day temperature conditions, the critical median inundation length needed for positive population growth is approximately 8 days. However, when egg survival decreases due to higher temperatures, this threshold increases to approximately 9 days. While this change might seem trivially small, it could have considerable consequences for long term persistence. In our study area, for example, this change would mean a 35% decline in the number of pools that can maintain fairy shrimp populations [29]; assuming that inundation lengths would remain unchanged from the present. In reality, however, it is unlikely that climate change will reduce egg survival without also altering inundation patterns, although it is unclear how exactly these will change. Some precipitation forecasts—but not all of them—suggest that early summer rains might increase as late summer rains decrease [31]. So, even if annual precipitation stays constant, it is likely that higher evaporation rates during late summer will accelerate the drying of pools and shorten the median length of inundations.

The long term growth rates of *B. wolfi* populations are highly dependent on long, but infrequent, inundation events [12], which can last for several months on Korannaberg [21]. As an explosive breeder the species benefits from such rare events by increasing the number of eggs in the propagule bank by several orders of magnitude. Considering that inundation lengths of any one pool are approximately log-normally distributed [29], such periods of rapid population expansion can help to compensate for the more frequent short inundations with low reproductive success. However, this compensatory mechanism is reliant on long term survival and staggered hatching of dormant eggs over multiple inundations.

In our experiment, *B. wolfi* egg hatching fractions were 12–22%. Partial hatching, with fixed hatching fractions proportionate to the probability of reproductive success, presumably evolved as part of a bet hedging strategy that buffers against unpredictable demographic catastrophes [11, 24, 32]. Our experiment showed that hatching fractions were unaffected by the long term exposure to increased temperatures. Delayed hatching is most successful as a bet hedging strategy when hatching fractions under suitable hatching conditions are consistent over time [7, 9]. Our results suggest that this is the case for *B. wolfi*. Although other studies have shown that the hatching success of dormant propagules can be affected by the storage temperature of eggs (see [18] for an overview), these did generally not distinguish whether such changes were due to an actual decrease in hatching fraction of viable eggs or reduced egg survival (see also [19]). We do, however, caution against generalizing the finding that hatching fraction is indifferent to higher temperatures. For instance, in their study of annual plants, Ooi and colleagues [15] found that higher temperatures affected the germination fractions of some species while the seeds of other species remained unaffected. Thus, a fruitful avenue for future research would be to examine how climate change affects the bet-hedging strategies of various species and how this might influence community interactions [33].

Our results showed that older *B. wolfi* eggs hatched more often than younger eggs. This might represent a ‘last-chance’ strategy to avoid mortality when energy reserves are almost depleted [18]. A mechanistic explanation for this pattern could also be that egg shell pigmentation degrades with time [34]. This means that *B. wolfi* embryos may become more receptive to light as an essential hatching cue [23] as they age. Alternatively, *B. wolfi* embryos might require variable amounts of time to complete development, which would mean that some of the younger eggs were not ready to hatch.

It should be noted that our study measured egg survival rates under laboratory conditions. In nature, survival rates should be considerably lower due to eggs being consumed by generalist predators, such as flatworms [35] and oribatid mites (unpublished data), blown away by strong winds [36] or washed away when ponds overflow [37]. Therefore, our calculations most likely overestimate future egg survival rates. Still, this does not necessarily mean that the studied populations would perish under climate change. For instance, predator and competitor populations may be affected by climate change as well, which would change biotic interactions. Next to that, vulnerability is also determined by the capacity of organisms to acclimate or adapt to novel environmental conditions [17, 38, 39]. Finally, spatial rescue-effects could reduce risks of, at least permanent, population extinction [22]. Although a sequence of short inundations in one pool could lead to a local extinction, it could be followed by recolonization from neighboring populations if these source populations are able to persist under climate change. Yet, in pools with median inundation lengths shorter than 9 days, the number of dispersing eggs required to maintain long term positive population growth under future conditions would be unrealistically high relative to field estimates of passive dispersal rates [36] as is discussed further in Additional file 5. Selection, followed by local adaptation, could also moderate the fitness effects of climate change [40] and a number of studies have picked up signatures of rapid selection for specific genotypes in response to increased temperatures in freshwater invertebrates [6, 41]. However, it seems as if *B. wolfi* populations are approaching their limits for adaptation because even under present-day conditions they cannot maintain permanently viable populations in the most ephemeral pools within the Korannaberg cluster. Future work should include experimental evolution trials during which zooplankton populations are exposed to realistic changes in temperature and inundation regime so that potential adaptation can be tracked across generations.

## Conclusions

Our study explored how life-history traits interact with environmental conditions to influence the demographics of temporary pool zooplankton species. While we integrate life-history responses and environmental changes, as expected under climate change, we need to combine this further with mechanistic aspects of evolution and species interactions to fully understand the consequences of climate change on aquatic biodiversity [39]. Nevertheless, our study is an important first step to understanding whether climate change can erode a species’ ability to withstand unfavorable habitat conditions. Our findings demonstrate clearly that climate change might not only jeopardize the persistence of aquatic species by reducing habitat suitability, but by also undermining the dormancy mechanism needed for demographic resilience.

## Additional files

**Additional file 1.** Study system and model species. Additional information and pictures on the study site and study organism.

**Additional file 2.** Daily temperature cycles. Temperature data and calculation of temperature cycles.

**Additional file 3.** Matrix population modelling. Additional information on the demographic matrix population modelling approach and additional results of the population modelling.

**Additional file 4.** Surviving and hatching fractions during the incubation experiment. Full overview of the results from the long term incubation experiment and overview of statistical model values.

**Additional file 5.** Dispersal and spatial rescue effects. Although not the main focus of this manuscript, dispersal can affect long term survival of the studied populations. To complement our study, we included a more elaborate discussion on the relevance of dispersal as a mediator of vulnerability under climate change in this additional file.
