# Supplementary material for: Climate change jeopardizes the persistence of freshwater zooplankton by reducing both habitat suitability and demographic resilience
Source: BMC Ecol. 2018 Jan 24;18:2. doi: 10.1186/s12898-018-0158-z (PMC5782365; doi:10.1186/s12898-018-0158-z)
Supplement: Supplementary file 1 — Additional file 1. Study system and model species. Additional information and pictures on the study site and study organism. [file 12898_2018_158_MOESM1_ESM.pdf]

Additional file 1: Study system and model species

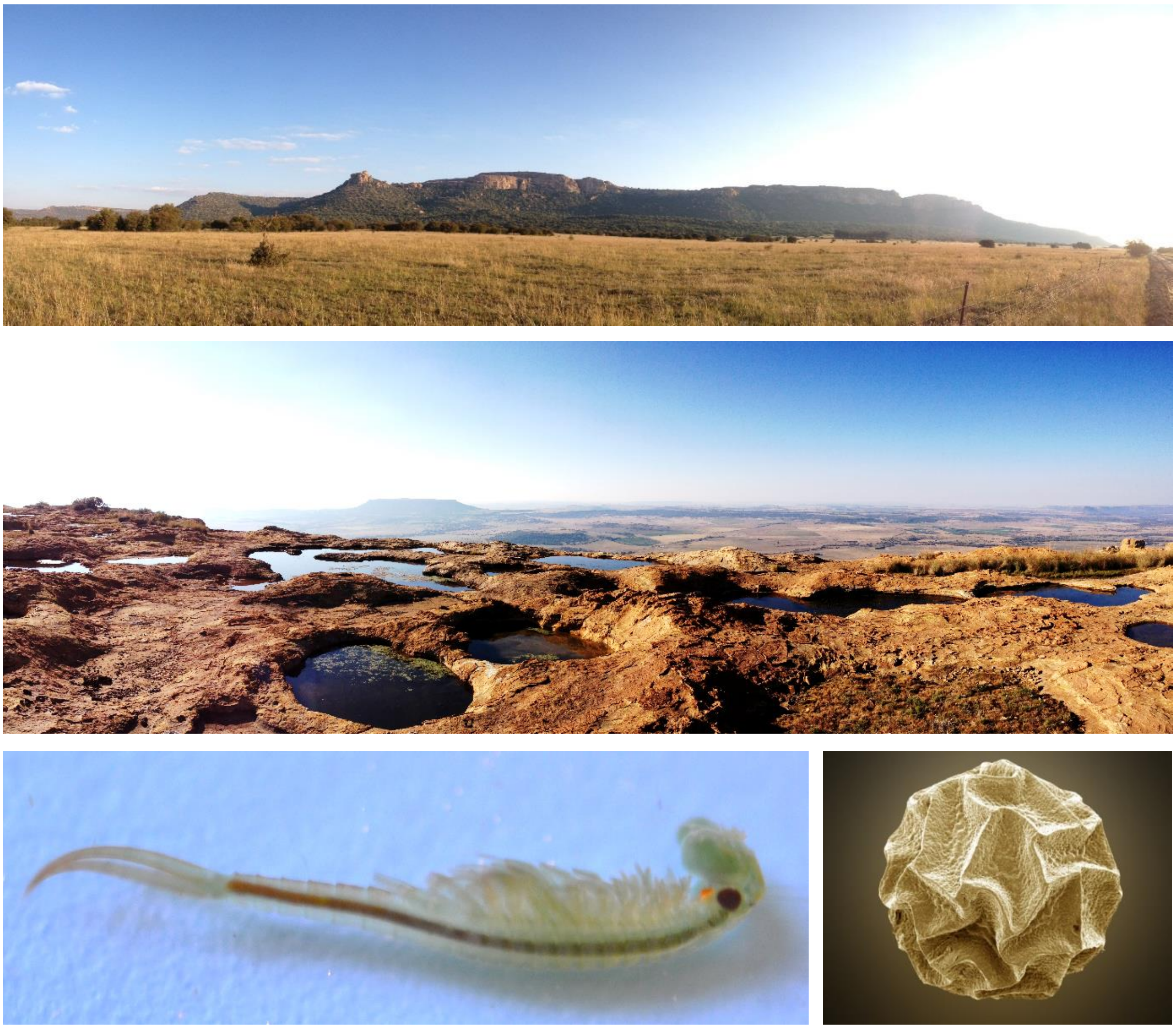

Additional Figure 1 Korannaberg mountain is a sandstone inselberg (top picture) in the Eastern Free State province in South Africa. On top of the inselberg, a number of basins have eroded that can temporarily hold water after rainfall until they dry-out due to evaporation (middle picture). Despite their temporary nature, these aquatic ecosystems are inhabited by a number of zooplankton species among which the fairy shrimp *Branchipodopsis wolfi* is most prevalent (bottom picture left, animal size: ± 2cm). Like many zooplankton species *B. wolfi* females produce drought resistant dormant eggs to bridge dry phases of their habitat (bottom picture right, egg size: ± 200µm).
