# Supplementary material for: Climate change jeopardizes the persistence of freshwater zooplankton by reducing both habitat suitability and demographic resilience
Source: BMC Ecol. 2018 Jan 24;18:2. doi: 10.1186/s12898-018-0158-z (PMC5782365; doi:10.1186/s12898-018-0158-z)
Supplement: Supplementary file 2 — Additional file 2. Daily temperature cycles. Temperature data and calculation of temperature cycles. [file 12898_2018_158_MOESM2_ESM.pdf]

Additional file 2: Daily temperature cycles

Additional Table 1 Daily temperature cycles as experienced by *Branchipodopsis wolfi* eggs in dry, sun-exposed rock pool sediment under present-day (Pres) and predicted future climate (Fut) conditions for the eight months of the incubation experiment. A conversion factor (C) was calculated to convert temperatures, as measured at the weather station, to temperatures in sun-exposed sediment.

		**October**		**November**		**December**		**January**		**February**		**March**		**April**		**May**	
**T(h)**	C	**Pres**	**Fut**	**Pres**	**Fut**	**Pres**	**Fut**	**Pres**	**Fut**	**Pres**	**Fut**	**Pres**	**Fut**	**Pres**	**Fut**	**Pres**	**Fut**
**0**	1.0	14.2	18.2	16.3	20.3	18.1	22.1	20.4	24.4	19.1	23.1	17.0	21.0	11.1	15.1	12.4	8.4
**1**	1.0	13.4	17.4	15.7	19.7	17.4	21.4	19.7	23.7	18.6	22.6	16.4	20.4	10.6	14.6	11.8	7.8
**2**	1.0	13.0	17.0	15.0	19.0	16.9	20.9	19.3	23.3	18.2	22.2	15.8	19.8	10.1	14.1	11.3	7.3
**3**	1.0	12.4	16.4	14.5	18.5	16.4	20.4	18.6	22.6	17.8	21.8	15.2	19.2	9.6	13.6	10.9	6.9
**4**	1.0	11.9	15.9	13.9	17.9	15.9	19.9	18.1	22.1	17.4	21.4	14.9	18.9	9.0	13.0	10.2	6.2
**5**	1.0	11.1	15.1	13.5	17.5	15.5	19.5	17.6	21.6	16.9	20.9	14.6	18.6	9.0	13.0	10.2	6.2
**6**	1.2	12.3	16.3	16.4	21.0	18.9	23.5	20.1	24.7	19.1	23.7	16.4	21.0	9.9	14.5	11.4	6.8
**7**	1.2	16.3	20.9	19.5	24.1	21.0	25.6	22.8	27.4	21.2	25.8	17.0	21.6	9.9	14.5	11.1	6.5
**8**	1.2	20.1	24.9	22.8	27.6	24.0	28.8	26.2	31.0	24.3	29.1	21.0	25.8	15.2	20.0	15.8	11.0
**9**	1.4	26.0	31.4	28.3	33.7	29.4	34.8	32.4	37.8	30.0	35.4	26.2	31.6	20.6	26.0	22.8	17.4
**10**	1.4	29.7	35.3	31.3	36.8	32.2	37.7	35.8	41.3	33.1	38.6	29.6	35.1	24.3	29.8	27.0	21.5
**11**	1.4	32.5	38.1	33.8	39.4	34.6	40.2	38.7	44.3	36.0	41.6	32.4	38.0	27.4	33.0	30.3	24.7
**12**	1.4	34.8	40.5	36.1	41.8	36.6	42.3	41.1	46.8	38.3	44.0	34.5	40.2	29.6	35.2	32.8	27.1
**13**	1.4	35.2	40.7	36.1	41.6	36.7	42.3	41.3	46.8	38.3	43.8	34.9	40.4	30.1	35.6	33.2	27.7
**14**	1.2	31.5	36.3	31.9	36.7	32.5	37.3	36.6	41.4	34.0	38.8	31.1	35.9	26.7	31.5	29.7	24.9
**15**	1.0	27.5	31.6	27.6	31.8	28.3	32.5	32.0	36.1	29.7	33.8	27.1	31.3	23.4	27.6	26.0	21.8
**16**	1.0	21.8	25.1	22.0	25.3	27.2	31.2	30.5	34.5	28.3	32.3	26.0	30.0	22.3	26.3	24.9	20.9
**17**	1.0	21.9	25.3	22.0	25.3	26.5	30.5	29.6	33.6	27.8	31.8	25.4	29.4	22.0	26.0	24.0	20.0
**18**	1.0	21.5	25.0	22.0	25.3	25.6	29.6	28.8	32.8	27.1	31.1	24.3	28.3	19.4	23.4	19.6	15.6
**19**	1.0	19.5	23.2	21.2	24.9	23.6	27.6	26.9	30.9	25.0	29.0	21.2	25.2	16.1	20.1	16.7	12.7
**20**	1.0	17.8	21.7	19.5	23.3	21.3	25.3	24.5	28.5	22.6	26.6	19.5	23.5	14.1	18.1	15.4	11.4
**21**	1.0	17.1	21.1	18.9	22.9	20.0	24.0	23.2	27.2	21.0	25.0	18.6	22.6	12.8	16.8	14.7	10.7
**22**	1.0	16.0	20.0	17.9	21.9	19.3	23.3	22.0	26.0	20.4	24.4	17.8	21.8	12.0	16.0	13.9	9.9
**23**	1.0	15.0	19.0	17.2	21.2	18.7	22.7	20.9	24.9	19.6	23.6	17.4	21.4	11.4	15.4	13.3	9.3
