# Supplementary material for: Climate change jeopardizes the persistence of freshwater zooplankton by reducing both habitat suitability and demographic resilience
Source: BMC Ecol. 2018 Jan 24;18:2. doi: 10.1186/s12898-018-0158-z (PMC5782365; doi:10.1186/s12898-018-0158-z)
Supplement: Supplementary file 3 — Additional file 3. Matrix population modelling. Additional information on the demographic matrix population modelling approach and additional results of the population modelling. [file 12898_2018_158_MOESM3_ESM.pdf]

Additional file 3: Matrix population Modelling

We re-parametrized a published matrix population model to investigate the impact of the measured changes in egg survival fraction on long term population growth rate and extinction probability (Additional Fig. 2, Additional Table 2, [1]). The model was initially parametrized in that paper with realistic estimates of present-day mean *B. wolfi* life history trait values, which were based on data from a large number of empirical studies [2-5]. For the purpose of the current study, we chose to retain all life history trait values, save for egg survival fractions, which were shown to be significantly affected by the future temperature treatment in our laboratory experiment. Egg survival (e_0 and e_1), was determined by calculating the mean survival across the four evaluation moments within each temperature regime. The life history trait values that were used for the present study can be consulted in Additional Table 2. Additionally, to explore a 'worst-case-scenario' we also ran the model with egg survival set to the lowest values measured within every treatment (Additional Fig. 3).

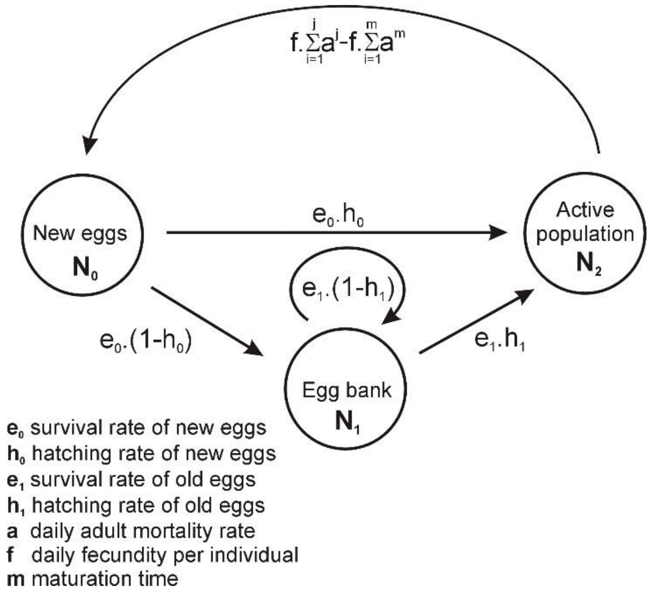

Additional Figure 2 Population dynamics of *Branchipodopsis wolfi* when inundations are long enough to allow maturation and reproduction. Three life stage classes are distinguished; young eggs that were produced during the previous inundation (N_0), older eggs (N_1) and individuals of the active population (N_2). Figure from [1].

Additional Table 2 *Branchipodopsis wolfi* life history trait values were obtained from the literature, field and laboratory experiments (see also [1]). The egg survival parameters were adjusted based on the results from our long term experiment (Additional Table 3).

Vital rate	Mean	Source
Egg survival:	e_0: 92 %<br>e_1: 88 %	Long term experiment (Constant 18 °C)<br>Long term experiment (Constant 18 °C)
Egg survival:	e_0: 88 %	Long term experiment (Present temperature cycle)
	e_1: 83 %	Long term experiment (Present temperature cycle)
Egg survival:	e_0: 85 %	Long term experiment (Future temperature cycle)
	e_1: 77 %	Long term experiment (Future temperature cycle)
Adult survival(a)	a: 74 %	[Unpublished data; L. Brendonck field observations]
Hatching fraction(h)	h_0: 47 %	[4,5,Unpublished data]
	h_1: 9 %	[4,Unpublished data]
Maturation time (m)	m: 6	[2,3,5]
Daily fecundity (f)	f: 13	[2,3]

The simulations were performed in R version 3.12 (R Development Core Team, Vienna, Austria, 2014) with starting values of N0 = N2 = 0 and N1 = 25000. Since inundation lengths can be highly variable in the investigated rock pools, we accounted for the impact of stochastic variation in the lengths of inundations on population dynamics, iterated our model 1000 times and based our conclusions on the average of these iterations. Each iteration ran over a sequence of 1100 inundations (each inundation time-step denoted by sub-script, *t*), with each inundation represented by a hydroperiod (length of inundation denoted by the sub-script *j*). The length of each hydroperiod, j, was simulated to match inundation lengths found in nature [6], by randomly drawing 1100 values from a log-normal distribution where the log-mean represented the log-median inundation length in days and the log-standard deviation was 1. By doing so, we were able to easily simulate a sequence of hydroperiods, with a pre-defined median length. Of the resulting sequence of 1100 independent inundations, most were short (<3 days); closely

resembling the frequency distribution of hydroperiods for mechanistically simulated inundations of the studied rock pools on Korannaberg [6,7].

For each iteration of our model, we simulated the population size of dormant eggs in the egg bank (N1) for every one of the 1100 time-steps (t). After allowing a burn-in period of 100 time-steps to account for transient dynamics based on the starting conditions, we calculated the stochastic population growth rate (logλ) as:

$$\log\lambda = \frac{1}{1000}\sum_{t=101}^{1100} \log\left(\frac{N_{1(t+1)}}{N_{1(t)}}\right)$$

If an inundation was long enough for hatched individuals to reach sexual maturity (m) and reproduce (i.e. m < j), we modelled changes as (1):

$$\begin{vmatrix} N_0 \\ N_1 \\ N_2 \end{vmatrix}_{t+1} = \begin{vmatrix} 0 & 0 & f.\sum_{i=1}^{j} a^j - f.\sum_{i=1}^{m} a^m \\ e_0.(1-h_0) & e_1.(1-h_1) & 0 \\ e_0.h_0 & e_1.h_1 & 0 \end{vmatrix} \times \begin{vmatrix} N_0 \\ N_1 \\ N_2 \end{vmatrix}_t \qquad (1)$$

Since survival and hatching rates differ between newly produced and old eggs, we distinguished between the egg survival (e) and hatching (h) rates of N_0 (e_0 and h_0, respectively) and N_1 (e_1 and h_1, respectively). The fairy shrimps only reproduce sexually so daily fecundity (f) for N_2 was averaged per breeding pair. Therefore, N_0 for any given inundation was the sum of the total fecundity on each day, considering that only a proportion of adults (a) survived to the next day. If the active population could not reach sexual maturity (m>j), population dynamics were modelled as (2):

$$\begin{vmatrix} N_0 \\ N_1 \\ N_2 \end{vmatrix}_{t+1} = \begin{vmatrix} 0 & 0 & 0 \\ e_0.(1-h_0) & e_1.(1-h_1) & 0 \\ e_0.h_0 & e_1.h_1 & 0 \end{vmatrix} \times \begin{vmatrix} N_0 \\ N_1 \\ N_2 \end{vmatrix}_t \qquad (2)$$

In case of negative long term growth rates, the probability of population extinction is 100 %. Yet, extinction is even possible with positive growth rates if the variance in short-term changes

in population size is high. We therefore estimated the probability of population extinction (E), according to Caswell [8], as (3):

$$E=\exp\left(\frac{-2.\log N_1(0).\log\lambda}{\sigma^2}\right)(3)$$

Where $N_1(0)$ is the starting number of dormant eggs in the egg bank.

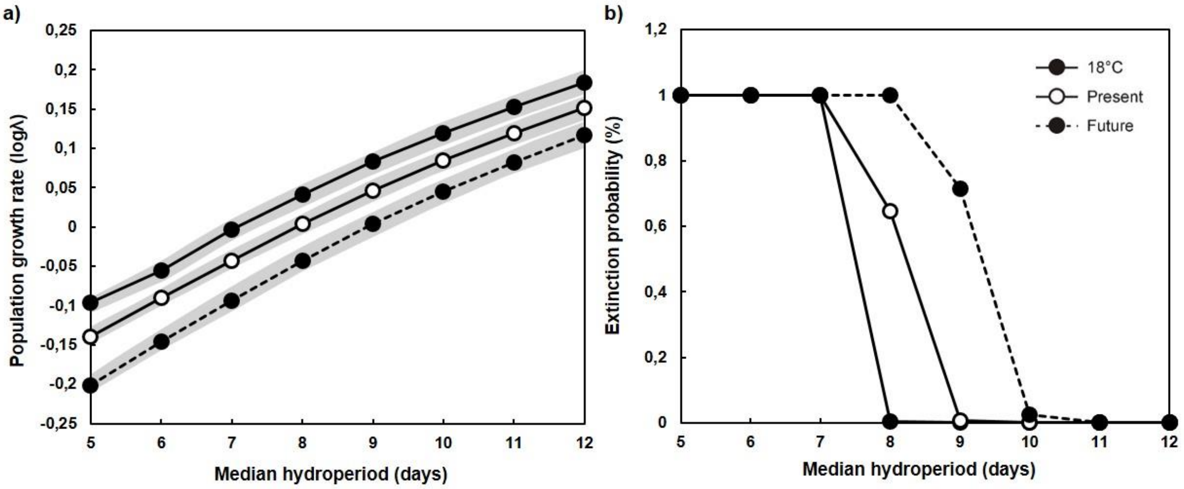

Additional Figure 3 Fractions of surviving *Branchipodopsis wolfi* eggs were determined during an eight month laboratory experiment under three different temperature treatments; a present-day cycle (Present), a future cycle (Future) and constant 18 °C (18 °C). When survival parameters of old and young eggs are set to the minimum measured values under the future temperature treatment, matrix population models indicate that **(a)** the median hydroperiod required for positive population growth increases and that also **(b)** the extinction risk of populations in pools with a certain median hydroperiod increases. The grey bands represent standard errors of population growth rate estimates.

References

1. Pinceel T, Vanschoenwinkel B, Brendonck L, Buschke F: Modelling the sensitivity of life history traits to climate change in a temporary pool crustacean. Sci. Reports 2016;6:29451.

2. Hamer ML, Appleton CC: Life-history adaptations of phyllopods in response to predators, vegetation, and habitat duration in Northeastern Natal. Hydrobiologia 1991;212:105-116.

3. Brendonck L, Riddoch B, Van de Weghe V: Hatching characteristics of the fairy shrimp Branchipodopsis wolfi in relation to the stochastic nature of its habitat, desert rock pools. Verhandlungen des internationalen Vereins für Limnologie 2001;27:3931-3935.

4. Vanschoenwinkel B. Dispersal, metapopulation-and metacommunity dynamics in a rock pool model system 2009;Phd Thesis.

5. Vanschoenwinkel B, Seaman MT, Brendonck L: Hatching phenology, life history and egg bank size of a fairy shrimp (Branchiopoda, Crustacea) in relation to the ephemerality of its habitat. Aquatic Ecol. 2010;44:771-780.

6. Tuytens K, Vanschoenwinkel B, Waterkeyn A, Brendonck L: Predictions of climate change infer increased environmental harshness and altered connectivity in a cluster of temporary pools. Freshwater Biol 2014, 59(5):955-968.

7. Vanschoenwinkel B, Hulsmans A, De Roeck ER, De Vries C, Seaman M, Brendonck L: Community structure in temporary freshwater pools: disentangling effects of habitat size and hydroregime Freshwater Biol. 2009;54:1487-1500.

8. Caswell H. Matrix population models. Wiley Online Library 2001.
