# Supplementary material for: Climate change jeopardizes the persistence of freshwater zooplankton by reducing both habitat suitability and demographic resilience
Source: BMC Ecol. 2018 Jan 24;18:2. doi: 10.1186/s12898-018-0158-z (PMC5782365; doi:10.1186/s12898-018-0158-z)
Supplement: Supplementary file 4 — Additional file 4. Surviving and hatching fractions during the incubation experiment. Full overview of the results from the long term incubation experiment and overview of statistical model values. [file 12898_2018_158_MOESM4_ESM.pdf]

Additional file 4: Surviving and hatching fractions during the incubation experiment

Our results demonstrated a significant decrease in the survival of both young and old *B. wolfi* eggs in the expected future climate compared to the present day temperature regimes. Consistent with our expectations, the measured survival fractions of both new and old eggs were markedly higher than the mean values under natural conditions established by Pinceel and co-workers [1]. This is due to the fact that, aside from the risk of dying during dormancy, eggs also face other risks in their natural environment. For instance, eggs may be lost due to egg bank erosion by wind action, during the dry phase [2]. In addition, during the wet phase, zooplankton eggs are known to fall victim to predators including mites, tadpole shrimps and flatworms [3,4]. Neither of these were taken into account in the performed experiment. Yet, we opted to use the values as measured in our experiment rather than an adjusted estimation to more directly link the *in silico* results of this experiment to population growth rate and extinction probability of *B. wolfi* populations.

Additional Table 3 Overview of *Branchipodopsis wolfi* surviving and hatching fractions (%) of old (aged twelve months at the start of the experiment) and young (aged two months at the start of the experiment) eggs. Survival and hatching fractions were determined every eight weeks throughout the course of the eight month incubation experiment. The experiment comprised three different temperature treatments; a present-day cycle (Present), an expected future cycle (Future) and constant 18 °C (18 °C). Both cycles represent daily temperature fluctuations that were calculated based on the average temperature for each hour of the day, with separate values calculated for each month of the year.

**Incubation Time**	**Egg Age**		**Survival**(%)			**Hatching**(%)	
		18 °C	Present	Future	18 °C	Present	Future
2 months	14 months	92.2	87.4	82.1	17.5	17.2	22.5
4 months	16 months	87.4	83.7	77.9	15.8	17.3	16.5
6 months	18 months	85.5	80.7	74.4	14.2	15.7	20.5
8 months	20 months	85.0	81.1	74.3	15.5	17.5	21.0
2 months	4 months	94.6	89.7	86.8	12.8	14.2	13.9
4 months	6 months	92.7	87.7	84.6	13.2	11.9	15.3
6 months	8 months	91.0	88.3	84.3	12.5	13.3	15.7
8 months	10 months	90.9	86.8	84.8	12.8	15.9	16.5

Additional Table 4 Estimated coefficients for the generalized linear mixed models investigating the impact of temperature treatment and incubation period on the surviving and hatching fraction of batches of 'old' and 'young' *Branchipodopsis wolfi* eggs. Survival and hatching fractions were determined every eight weeks throughout the course of the eight month incubation experiment. The experiment comprised three different temperature treatments; a present-day cycle (Present), an expected future cycle (Future) and constant 18 °C (18 °C). Both cycles represent daily temperature fluctuations that were calculated based on the average temperature for each hour of the day, with separate values calculated for each month of the year.

a) Surival	Estimate	St. Error	z value
**Intercept**	2.803	0.149	18.850
**Temperature**	-0.733	0.114	-6.416
**Incubation period**	-0.017	0.005	-3.421
**Egg age (Old)**	-0.484	0.091	-5.288
**b) Hatching**	**Estimate**	**St. Error**	**z value**
**Intercept**	-1.953	0.140	-13.927
**Temperature**	0.262	0.114	2.300
**Incubation period**	0.001	0.005	0.164
**Egg age (Old)**	0.276	0.094	2.940
