# Supplementary material for: Climate change jeopardizes the persistence of freshwater zooplankton by reducing both habitat suitability and demographic resilience
Source: BMC Ecol. 2018 Jan 24;18:2. doi: 10.1186/s12898-018-0158-z (PMC5782365; doi:10.1186/s12898-018-0158-z)
Supplement: Supplementary file 5 — Additional file 5. Dispersal and spatial rescue effects. Although not the main focus of this manuscript, dispersal can affect long term survival of the studied populations. To complement our study, we included a more elaborate discussion on the relevance of dispersal as a mediator of vulnerability under climate change in this additional file. [file 12898_2018_158_MOESM5_ESM.pdf]

Additional file 5: Dispersal and spatial rescue effects

Dispersal of *B. wolfi* eggs is a passive process which is predominantly dependent on wind and water as vectors. Theoretically, influx of *B. wolfi* eggs via dispersal could compensate for negative population growth or could lead to recolonization after a population goes extinct. Still, our matrix population models indicate that the number of dispersing eggs that would be required to maintain long term positive population growth in pools with a median inundation length shorter than nine days is unrealistically high. For instance, if egg survival rates were set to the average values that were measured in the future temperature treatment, population growth in a pool with a median inundation length of eight days would be -2.3 % (compared to 2.2 % in the present day temperature treatment). This means that, just in order to compensate for increased egg mortality under increased temperatures, 4.5 % of the total number of eggs in the egg bank would need to be replenished after every inundation via dispersal. Since population sizes of the studied pools range between 6 x 10^3 and 5.6 x 10^6 [1], in between 270 and 252000 eggs would need to be added to the egg bank via dispersal over the course of each dry season. Field studies, during which dispersal of *B. wolfi* eggs was quantified in the studied cluster, suggest that these numbers far exceed actual dispersal.

Wind-dispersed *B. wolfi* eggs were intercepted on glue surfaces in the studied rock pool cluster to quantify dispersal rates among pools. Over the course of one month, 40 *B. wolfi* eggs were counted per m^2 within the pool cluster with outliers of up to 300 eggs per m^2 in the immediate proximity of large source habitats [2]. These rates, however, were obtained at a time when most eggs are available for dispersal and dispersal fluxes are maximal since the pools had all just dried after a period of reproduction. Also, it must be noted that not all eggs that reach another pool basin at some point during the dry phase would actually stay there until the next growing season. Winds can pick them up again and blow them from the basin. Aside from wind, flowing water is a dominant dispersal vector for invertebrates among the studied pools. Some

pools temporarily overflow after heavy rainfall and eggs can be transported from one pool basin to another during such sporadic floods. Compared to wind, an advantage of this dispersal mode is that eggs end up in a wet pool basin and are more likely to dry into the sediment and remain fixated there until the next inundation. Based on direct measurements, a pool can receive between 40 and 3000 eggs via such overflows per year. Such overflow connections, however, are quite rare and only occur between a limited number of pools. These estimates of wind and water mediated dispersal do not take into account that climate change will ensure that less eggs will be available for dispersal within the metapopulation. Combined with the fact that eggs may be predated upon and that arriving eggs may be blown out of basins again, this implies that dispersal is unlikely to provide sufficient eggs to maintain long term population growth under climate change.

References

1. Vanschoenwinkel B, Seaman MT, Brendonck L: Hatching phenology, life history and egg bank size of a fairy shrimp (Branchiopoda, Crustacea) in relation to the ephemerality of its habitat. Aquatic Ecol. 2010;44:771-780.

2. Vanschoenwinkel B, Hulsmans A, De Roeck ER, De Vries C, Seaman M, Brendonck L: Community structure in temporary freshwater pools: disentangling effects of habitat size and hydroregime Freshwater Biol. 2009;54:1487-1500.
